# Mapping Disorder in Polycrystalline Relaxors: A Piezoresponse Force Microscopy Approach

**DOI:** 10.3390/ma3114860

**Published:** 2010-10-28

**Authors:** Andrei L Kholkin, Dmitry A Kiselev, Igor K Bdikin, Andris Sternberg, Brahim Dkhil, Stephen Jesse, Oleg Ovchinnikov, Sergei V Kalinin

**Affiliations:** 1 Department of Ceramics and Glass Engineering & CICECO, University of Aveiro, 3810-193 Aveiro, Portugal; E-Mail: dmitry@ua.pt (D.A.K.); 2 Department of Mechanical Engineering & TEMA, University of Aveiro, 3810-193 Aveiro, Portugal; E-Mail: bdikin@ua.pt (I.K.B.); 3 Institute of Physics of the Latvian Academy of Sciences, Kengaraha st., LV-1063 Riga, Latvia; E-Mail: stern@latnet.lv (A.S.); 4 Laboratory Structures, Propriétés et Modélisation des Solides, Ecole Centrale Paris, CNRS, UMR 8580, Grande Voie des Vignes, F-92295 Chatenay-Malabry Cedex, France; E-Mail: brahim.dkhil@ecp.fr (B.D.); 5 The Center for Nanophase Materials Sciences, Oak Ridge National Laboratory, Oak Ridge, TN 37922, USA; E-Mails: sjesse@ornl.gov (S.J); ovchinnikov1@ornl.gov (O.O.); sergei2@ornl.gov (S.V.K.)

**Keywords:** PLZT, relaxors, Piezoresponse Force Microscopy, domains, grains

## Abstract

Relaxors constitute a large class of ferroelectrics where disorder is introduced by doping with ions of different size and valence, in order to maximize their useful properties in a broad temperature range. Polarization disorder in relaxors is typically studied by dielectric and scattering techniques that do not allow direct mapping of relaxor parameters, such as correlation length or width of the relaxation time spectrum. In this paper, we introduce a novel method based on measurements of local vibrations by Piezoresponse Force Microscopy (PFM) that detects nanoscale polarization on the relaxor surface. Random polarization patterns are then analyzed via local Fast Fourier Transform (FFT) and the FFT PFM parameters, such as amplitude, correlation radius and width of the spectrum of spatial correlations, are mapped along with the conventional topography. The results are tested with transparent (Pb, La) (Zr, Ti)O_3_ ceramics where local disorder is due to doping with La^3+^. The conclusions are made about the distribution of the defects responsible for relaxor behavior and the role of the grain boundaries in the macroscopic response.

## 1. Introduction

Relaxor ferroelectrics are probably the most interesting and mysterious objects of solid state physics studied over the last 50 years. In contrast to conventional ferroelectrics whose ferroelectric, dielectric and piezoelectric properties are described by the thermodynamic theory, relaxor ferroelectrics (or simply relaxors) possess a number of unique (and yet not fully explored) properties that make them promising candidates for numerous applications such as piezoelectric actuators, multilayer capacitors, tunable filters, *etc*. In particular, they exhibit high and diffuse dielectric permittivity (*i.e*., without distinct peak at the transition point, T_c_), absence of both macroscopic spontaneous polarization and symmetry distortion below the peak, and pronounced non-ergodicity and glass-like properties at low temperatures [1]. However, if a sufficiently strong electric field is applied, the ferroelectric phase may develop extraordinary piezoelectric properties, especially in solid solutions with normal ferroelectrics [2,3]. Two essential features are well recognized in relaxors: (i) pronounced disorder due the aliovalent (causing random electric fields) or isovalent (creating random stress fields) doping, (ii) appearance of small ordered nanoregions (polarization clusters) far above T_c_. These polarization clusters arise at high temperature (called Burns temperature) and develops into “normal” macroscopic ferroelectric domains only when cooled under sufficiently high electric field. It is the dynamics of these clusters that causes the peculiar dielectric and piezoelectric behavior of relaxors. Until recently, the properties of polar clusters in relaxors could only be assessed via indirect techniques such as x-ray, electron, or neutron diffraction, where dynamical properties of these clusters contribute to diffuse scattering parameters such as pair correlation function. The size of these regions can be roughly estimated via the digital processing of electron diffraction patterns that show doubling of the unit cell due to the short-range order appearing at low temperature. However, no technique existed until recently that allowed direct mapping of the mesoscopic disorder and evaluation of the parameters of the disorder such as correlation radius and width of the relaxation time spectrum.

Recently developed Piezoresponse Force Microscopy (PFM) [4,5] is used in this work to image not only peculiar polarization patterns in relaxor ferroelectrics but also, after computer processing, to map their major parameters with the sub-μm resolution. It is believed that such studies can shed light on the nature of polarization disorders and the role of mesoscale defects, e.g., grain boundaries or built-in electric fields associated with charged defects.

## 2. Experimental Details

In this work, we studied Pb_0.9125_La_0.0975_(Zr_0.65_Ti_0.35_)_0.976_O_3_ ceramics (PLZT 9.75/65/35) of the composition which is (on average) in the non-ergodic relaxor state with the temperature of the maximum of the dielectric permittivity of 340 K and freezing temperature 296 K. The samples were prepared by the well known two-stage sintering method, where the corresponding powders (obtained by chemical precipitation) were cold-pressed and first sintered in vacuum at 950–970 °C for 1 hour. Then they were pressure sintered (200 MPa) at 1150–1200 °C for 4 hours. The samples were cooled down to room temperature, cut and polished to optical quality. Further annealing at 600° was done to relieve the mechanical stress induced by polishing.

To analyze the image texture, 128 × 128 pixel sub-image centered at $\left(x_{1},y_{1}\right)=\left(64,64\right)$ was chosen in the corner of the 3,000 × 3,000 full image. The 2D FFT transform is performed within the selected region, and resulting 2D data set is fitted using phenomenological function $Z_{2D}$ as described below. Here, the function is chosen so as to reproduce the central peak and the ring, and allow for texture/preferential orientations (*i.e*., cross-section is ellipse rather then rotationally invariant). After fitting, the sub-image is shifted by 64 points, and the fitting was repeated. The fitting parameters are plotted as 2D maps for Mod(3,000/64) = 46 points. This process thus allows extended 2D version of analysis in [6]. Note that adjacent points are not independent [since sub-images overlap by half], but the next nearest neighbors are independent. Hence, gradients in the texture images are gradual if spanning more than 2 adjacent pixels.

The phenomenological fitting function is

(1) $$Z_{2D}=Z_{e}+Z_{c}+Z_{0},$$

where $Z_{e}$ and $Z_{c}$ are ellipsoidal ring and central peak contributions respectively, and $Z_{0}$ is position-independent offset. The functions

(2) $$Z_{e}=A_{1}\text{exp}(-\frac{{\left(a-d\right)}^{2}}{s_{1}^{2}}),$$

(3) $$Z_{c}=A_{2}\text{exp}(-\frac{b^{2}}{s_{2}^{2}}),$$

where $A_{1}$ and $A_{2}$ are the intensities of the texture and central peak, d is the characteristic wavevector of the domain, and $s_{1}$ and $s_{2}$ are corresponding widths. The coordinates are defined as

(4) $$a^{2}=x_{2}^{2}+{\left(y_{2}/\left(1-\varepsilon_{1}\right)\right)}^{2},$$

(5) $$b^{2}=x_{2}^{2}+{\left(y_{2}/\left(1-\varepsilon_{2}\right)\right)}^{2},$$

where $\varepsilon_{1}$ and $\varepsilon_{2}$ are eccentricities. Finally, $x_{2}=x\text{cos}\varphi +y\text{sin}\varphi$ and $y_{2}=-x\text{sin}\varphi +y\text{cos}\varphi$, where *x*, *y* correspond to the components of wave vector and *φ* is rotation angle. The maps of nine independent parameters (two amplitudes, two eccentricities, two widths, radius, angle, offset) are plotted as 2D maps.

## 3. Results and Discussion

**Figure 1:**
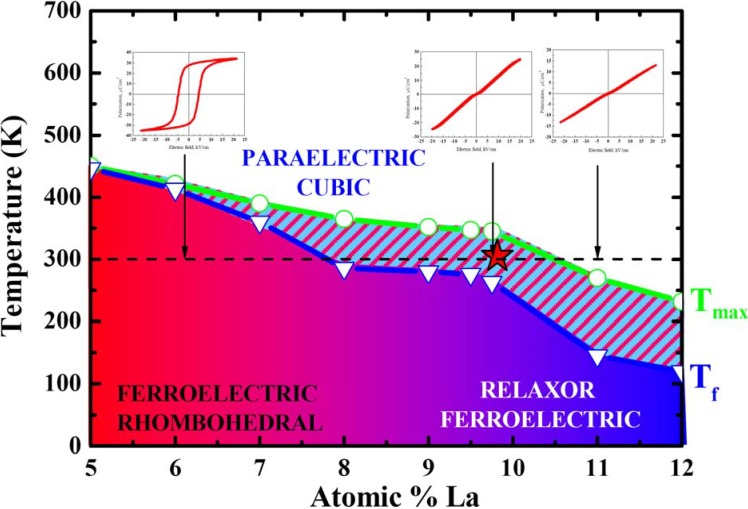
Phase diagram of the PLZT x/65/35 ceramics. *P-E* hysteresis loops of the PLZT *x*/65/35 ceramics (for *x* = 6, 9.75 and 13 La mole per cent) measured at room temperature.

As a model system for the polarization disorder mapping, we have chosen transparent Pb_1-x_La_x_(Zr_y_Ti_1-y_)_1-x/4_O_3_ ceramics that have been an object of intensive investigations since the discovery of its useful ferroelectric, piezoelectric, electrostrictive and electrooptic properties at the end of the 1960s [7]. The addition of La^3+^ to the conventional PZT ceramics leads to enhanced densification rates, more homogeneous microstructures and, as a result, to a high transparency in the ceramics prepared using hot pressing. In addition, it has allowed rich phase diagram to be achieved (Figure 1), with a continuous transition from typical ferroelectric/piezoelectric to relaxor/electrostrictive behavior.

Softening of the material is accompanied with the reduction of the transition point leading to high dielectric and electrooptic properties at room temperature. In 1972, Haertling and Land [8] patented the process of manufacturing transparent PLZT ceramics using chemical route and found that thus sintered ceramics have unique electrooptic properties ranging from linear (Pockels) effect at low La content to quadratic (Kerr) behavior. The discovery of PLZT has led to many applications including eye protective goggles for U.S. army (Sandia) and image storage devices and viewfinders (Sony). Aliovalent La^3+^ ions substitute Pb^2+^ and thus create charge imbalance due to vacancies in both A- and B-sites of the perovskite lattice. These defects are responsible for random electric and stress fields that destroy long-range ferroelectric order and result in the formation of polarization clusters. These clusters create inhomogeneous piezoelectric contrast on the surface of relaxor PLZT (x/65/35) previously observed by several groups [6,9,10]. It has been recently shown that the piezoelectric contrast and configuration of nanodomains depend on the local position of the PFM tip in the grain, thus enabling understanding of apparent grain size effect in relaxors [11]. The reduction of the correlation length near the grain boundary could arise due to either compressive stress concentration that increases disorder and shifts the transition point [1] or to the segregation of La at the grain boundaries [12].

**Figure 2:**
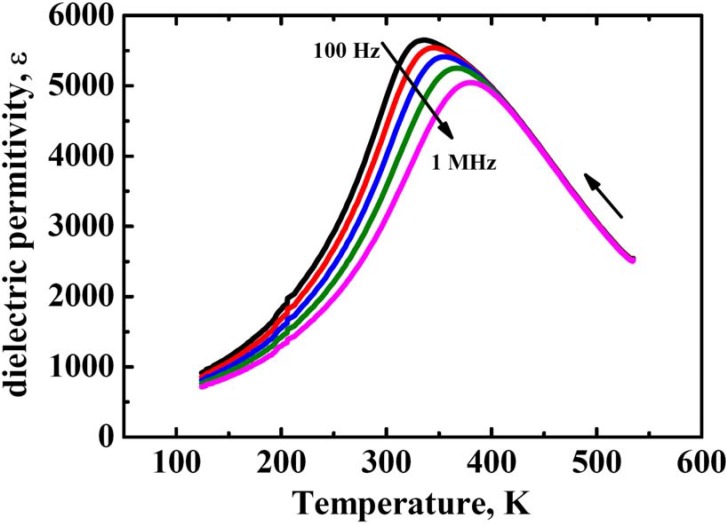
Temperature dependence of dielectric permittivity ε(T) of PLZT-9.75/65/65 ceramics, measured at different frequencies.

The dielectric properties of ceramics (Figure 2) demonstrate typical relaxor behavior with the temperature transition between relaxor ergodic and non-ergodic phases close to room temperature. It has been reported that the inhomogeneity of La^3+^ (as well as corresponding vacancies) distribution still limits the application of electrooptic PLZT ceramics, reducing its transparency to 65–70%. It is obvious that inhomogeneous distribution of defects in PLZT leads to inhomogeneous distribution of the major relaxor parameters such as correlation length, freezing temperature, width of the relaxation time spectrum, *etc*.

**Figure 3:**
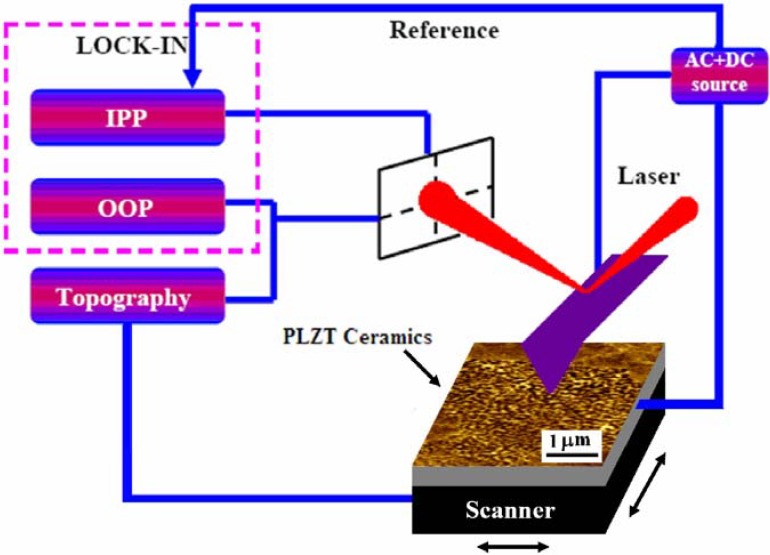
Schematic of the observed nanodomains (beneath the surface) and the configuration of the PFM measurements where the voltage is applied via a grounded conducting tip.

The typical domain pattern is shown in Figure 3 which also illustrates the schematic of the observed nanodomains (beneath the surface) and the configuration of the PFM measurements where the voltage is applied via a grounded conducting tip.

**Figure 4:**
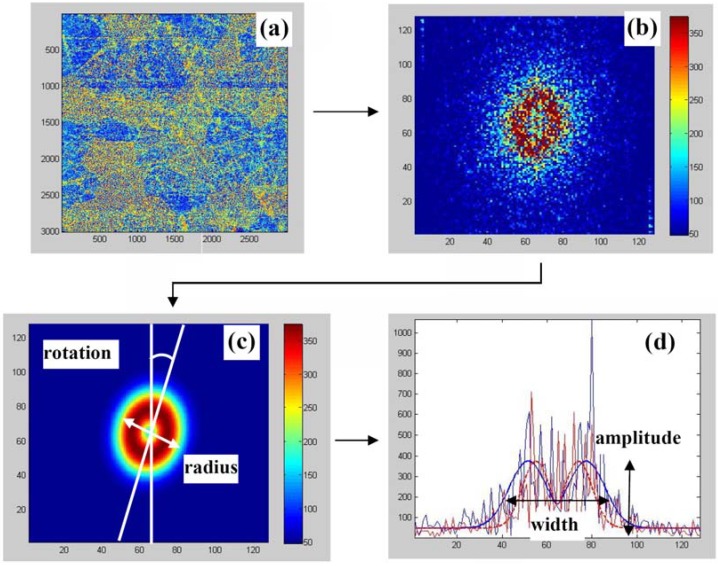
The interface (step-by-step) of a program for plotting maps images after 2D-FFT procedure. (**a**) piezoresponse image of PLZT ceramics; (**b**) Fast Fourier Transform (FFT); (**c**) FFT after smoothing; (**d**) cross-section from (**c**).

Details of the PFM instrumentation can be found elsewhere [13]. In order to analyze the distribution of nanodomains over the area containing several grains, we performed large scans (30 × 30 μm^2^ with the resolution 3,000 × 3,000 pixels) (Figure 4) and made Fast Fourier Transform (FFT) analysis in windows containing 128 × 128 pixels. Since the pixel size (10 × 10 nm^2^) is much smaller than the domain size, each window reproduces domain distribution with sufficiently high accuracy. On the other hand, there are enough features in the selected windows for the FFT analysis.

**Figure 5:**
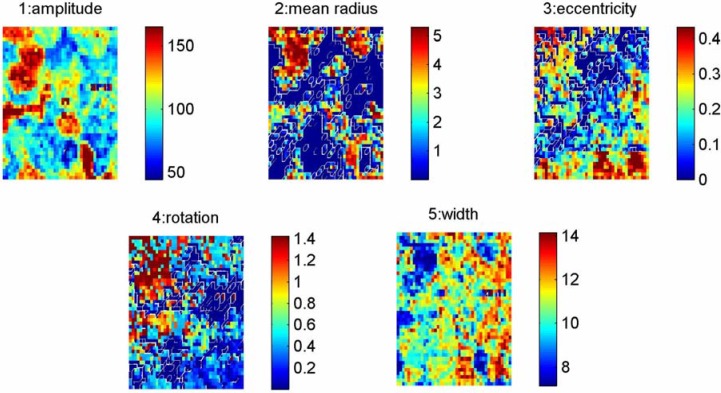
Represents an example of the FFT processing of the selected windows where different parameters such as, 1. amplitude, 2. radius, 3. eccentricity, 4. rotation and 5. width of the spatial correlation spectrum, could be extracted.

**Figure 6:**
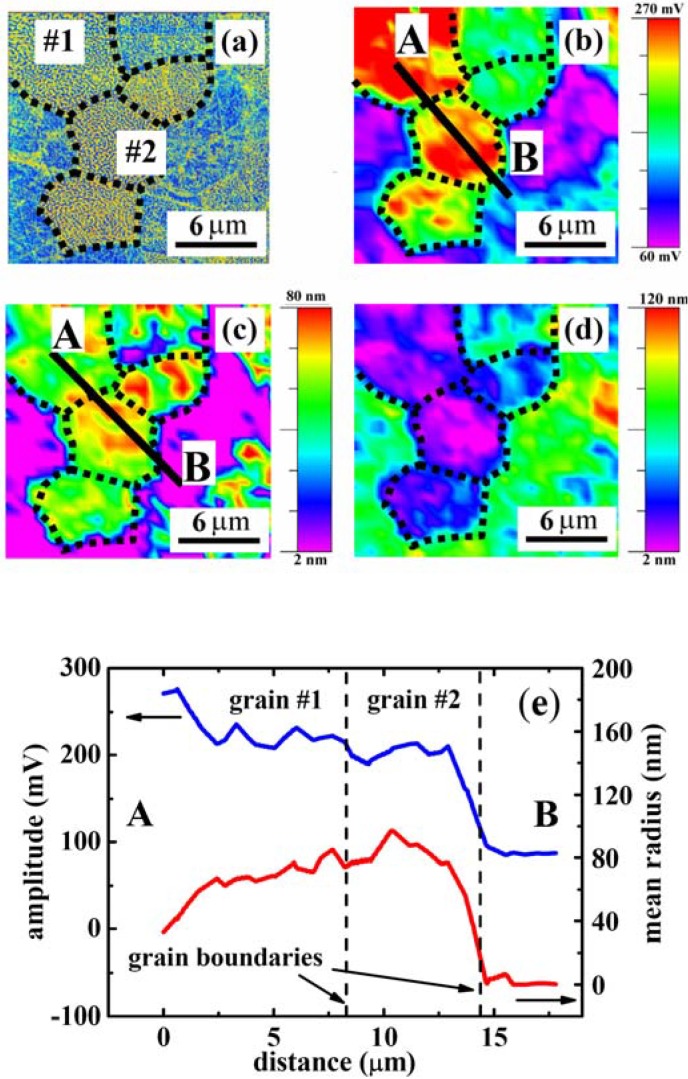
Original phase image (**a**) of the contrast grain, maps of the amplitude (**b**); the radius (**c**) and the width (**d**) after FFT procedure; (**e**) variation of the piezoresponse (blue line) and radius (red line) across of two neighboring grains (for **b**,**c**).

Figure 5 represents an example of the FFT processing of the selected windows where different parameters such as amplitude, radius, eccentricity, rotation and width of the spatial correlation spectrum could be extracted. The results are shown in Figure 6 together with original phase image of the contrast and grain boundaries denoted by dashed lines.

The microscopic mechanism involved in relaxors is still puzzling as it is difficult to deal with the nanoscale inhomogeneities whose observation depends on the length and time scale of the experimental probe. Besides, it is believed that the properties cannot be explained without considering hierarchical structures and dynamics underlying the need of multiscale tools ranging from atomic scale to large ferroelectric domains.

Figure 6 is the first attempt to map different parameters of the polarization distribution obtained while observing the microstructure of ceramics (visible grains in Figure 6(a)) after our FFT procedure. Among the parameters we could extract, the amplitude (Figure 6(b)), the radius (Figure 6(c)) and the width of the spatial spectrum (Figure 6(d)), are of great importance as new features can be revealed because they correspond to the average piezocoefficient—or, in other words, the polarization magnitude, the correlation length, and the distribution of the characteristic length scales, respectively. These pictures complement classical maze-like domain images obtained by PFM (Figure 6(a)) in relaxor materials, and show clearly that this method provides a powerful and unique tool for investigation of inhomogeneous or disordered materials at the mesoscale. It is worth noting that, whereas the classical PFM phase displays polarization inhomogeneities at the nm scale, the 2D-FFT processing provides information at an upper (meso)scale, as the inhomogeneities revealed with PFM phase is not more evidenced and, instead, new and larger sized features are seen.

Let us start by looking at the amplitude map that concerns the strength of the polarization within a several microns grain size. As one can see, some submicron-sized clusters randomly distributed within one grain are superimposed on the maze polarization usually observed by PFM phase. It is worth mentioning that the polarization adapts itself close to the grain boundaries. This could be explained either by stress accommodation or pinning through defects (most probably La^3+^). This picture visualizes the value of polarization (not well seen on classical PFM images) and reflects the role of the grain boundaries in the development of polarization. Some grain boundaries (probably with low angle between rhombohedral axes) do not affect the polarization and thus can be distinguished from high angle grain boundaries where the polarization is strongly disrupted.

The spatial inhomogeneities within the grain can be seen as a mixture of polar regions with some sort of glassy correlations, in agreement with earlier suggestions [14,15,16]. Indeed, the red zones (in the bottom grain or grain 2) indicate the strongest polarization amplitude. However, the correlation length associated with these polarization regions is relatively weak as one can judge from the map of correlation length (Figure 6(c)). This situation can be considered as the existence of a core of polarizations with all of them being correlated (mean correlation length) and embedded as a core-shell with a more randomly distributed or glassy-like polarization. This is well presented on the cross-section showing similar behavior of the amplitude and correlation radius at the grain boundary (Figure 6(e)).

It is interesting that the described regions show a rather small width indicating a narrow distribution of length scales and, as a consequence, of relaxation times. A narrow width is also measured in the top grain (grain 2). In this latter grain, the correlation length is rather big as its value is around 60 nm on average. This situation can be interpreted by considering strong enough interacting polarizations with collective dynamics. Such an interpretation can also be used for the description of bottom grain (grain 1). Moreover, interestingly, the shape of the correlation length of the top grain is very similar to that observed with PFM phase but on another scale. This is typical of a fractal behavior. It is remarkable that inside grain 2, the contour of the increased radius (higher correlation length or average domain size) fully corresponds to the distribution of the width (Figure 6(d)), *i.e*., the width of the spectrum of spatial correlations or roughly the spectrum of relaxation times. The measure of the width of the correlation spectrum is so-called freezing temperature T_f_ at which the relaxation spectrum becomes infinitely broad. The rough correspondence of the two parameters (T_f_ and R_c_) reflects the closeness of the selected composition to the non-ergodic state where local change of the correlation length is immediately translated into the variation of T_f_. We therefore argue that presented maps are the true images of the relaxation parameters of the relaxor PLZT near the surface. In this context, we should point out that the correlation radius evaluated by FFT is several times greater than that measured by TEM [17]. This can be attested to the influence of the surface as the PFM is sensitive to the piezoelectric vibrations in the layer 50–100 nm deep into the material. The origin of the spatial variation of the relaxor parameters in PLZT can be understood as follows. La^3+^ ion substitutes for Pb^2+^ ions in the PZT lattice, and because it possesses a larger positive charge compared with the host ion, it creates charge imbalance that depends on volatility of PbO during sintering. If there is PbO loss defect chemistry predicts reduction of oxygen vacancies in the lattice and increase of resistivity. If the PbO loss is not allowed (sintering in a PbO atmosphere), it favors creation of Pb vacancies as the compensating species. In this scenario there is no change in the resistivity, but dipolar defects can be easily formed. Typically, both scenarios coexist because it is difficult to prevent evaporation of PbO during liquid sintering. The ionic defects such as Pb and O vacancies can agglomerate [18] and form defect clusters near which local random electric fields may disrupt long-range polarization order and create isolated polarization clusters whose statistical properties reflect the distribution of such defects. It is therefore possible that the distribution of relaxor parameters reflects the distribution of random electric fields and thus defect accumulation near the surface.

## 4. Conclusion

In conclusion, we developed a novel method for testing ferroelectric disordered materials that allows for direct mapping of the relaxation parameters such as average polarization, correlation length and width of the spectrum of relaxation times. The method is tested with transparent PLZT ceramics widely used for electrooptic and actuator applications. The analysis shows that mapping of the relaxor parameters is useful in order to understand the role of grain boundaries and inhomogeneous defect distribution in the overall macroscopic properties of these materials.

## References

[1] Samara G.A. (2003). The relaxational properties of compositionally disordered ABO_3_ perovskites. J. Phys. Cond. Matt..

[2] Kuntjak Z., Petzelt J., Blinc R. (2006). The giant electromechanical response in ferroelectric relaxors as a critical phenomenon. Nature.

[3] Raevskaya S.I., Emelyanov A.S., Savenko F.I., Panchelyuga M.S., Raevski I.P., Prosandeev S.A., Colla E.V., Chen H., Lu S.G., Blinc R., Kutnjak Z., Gemeiner P., Kamzina L.S. (2007). Quasivertical line in the phase diagram of single crystals of PbMg_1∕3_Nb_2∕3_O_3_-xPbTiO_3_ (x = 0.00, 0.06, 0.13, and 0.24) with a giant piezoelectric effect. Phys. Rev. B.

[4] Kalinin S.V., Setter N., Kholkin A.L. (2009). Electromechanics on the nanometer scale: Emerging phenomena, devices, and applications. MRS Bull..

[5] Wu A., Vilarinho P.M., Shvartsman V.V., Suchaneck G., Kholkin A.L. (2005). Domain populations in lead zirconate titanate thin films of different compositions via piezoresponse force microscopy. Nanotechnology.

[6] Kiselev D.A., Bdikin I.K., Selezneva E.K., Bormanis K., Sternberg A., Kholkin A.L. (2007). Grain size effect and local disorder in polycrystalline relaxors via scanning probe microscopy. J. Phys. D..

[7] Haertling G.H., Levinson L.M. (1988). Electro-optic ceramics and devices. Electronic Ceramics.

[8] Haertling G.H., Land C.E. (1972). Recent improvements in the optical and electrooptic properties of PLZT ceramics. Ferroelectrics.

[9] Shvartsman V.V., Kholkin A.L., Orlova A., Kiselev D., Bogomolov A.A., Sternberg A. (2005). Polar nanodomains and local ferroelectric phenomena in relaxor lead lanthanum zirconate titanate ceramics. Appl. Phys. Lett..

[10] Nikolaeva E.V., Shur V.Y., Shishkin E.I., Sternberg A. (2006). Nanoscale domain structure in relaxor PLZT x/65/35 ceramics. Ferroelectrics.

[11] Okazaki K., Nagata K. (1973). Effects of grain size and porosity on electrical and optical properties of plzt ceramics. J. Am. Cer. Soc..

[12] Ling W.K., Chang Y.H. (1989). Properties and microstructures of PLZT ceramics hot-pressed from commercial powders. Ferroelectics.

[13] Kholkin A.L., Kalinin S., Gruverman A. (2006). Scanning Probe Microscopy: Electrical and Electromechanical Phenomena at the Nanoscale.

[14] Blinc R., Laguta V., Zalar B. (2003). Field cooled and zero field cooled 207Pb NMR and the local structure of relaxor PbMg_1/3_Nb_2/3_O_3_. Phys. Rev. Lett..

[15] Jeong I.K., Darling T.W., Lee J.K., Proffen T., Heffner R.H., Park J.S., Hong K.S., Dmowski W., Egami T. (2005). Direct observation of the formation of polar nanoregions in Pb(Mg_1/3_Nb_2/3_)O_3_ using neutron pair distribution function analysis. Phys. Rev. Lett..

[16] Colla E.V., Vigil D., Timmerwilke J., Weissman M.B., Viehland D.D., Dkhil B. (2007). Stability of glassy and ferroelectric states in the relaxors PbMg_1∕3_Nb_2∕3_O_3_ and PbMg_1∕3_Nb_2∕3_O_3_-12% PbTiO_3_. Phys. Rev. B.

[17] Viehland D. (1993). Origin of F spots and stress sensitivity in lanthanum lead zirconate titanate. J. Appl. Phys..

[18] Dawber M., Scott J. (2000). A model for fatigue in ferroelectric perovskite thin films. Appl. Phys. Lett..

